# Effects of Plantar Flexor Stretching on Static and Dynamic Balance in Healthy Adults

**DOI:** 10.3390/ijerph20021462

**Published:** 2023-01-13

**Authors:** Eui-Young Jung, Jin-Hwa Jung, Hwi-Young Cho, Sung-Hyeon Kim

**Affiliations:** 1 Department of Health Science, Gachon University Graduate School, Incheon 21936, Republic of Korea; 2 Department of Occupational Therapy, Semyung University, Jecheon 27136, Republic of Korea; 3 Department of Physical Therapy, Gachon University, Incheon 21936, Republic of Korea

**Keywords:** balance, static stretching, dynamic stretching, ballistic stretching

## Abstract

Stretching can affect balance ability by generating biomechanical and physiological changes in the postural muscles. Stretching of the lower extremity muscles can greatly affect posture maintenance strategies and balance ability. However, the relationship between stretching and balance ability has not been clarified. Therefore, this study aimed to investigate the effect of plantar flexor stretching on balance ability. Forty-four healthy young adults were randomly assigned to four groups (static stretching, dynamic stretching, ballistic stretching, and control). Ankle joint range of motion, static balance ability, and dynamic balance ability were evaluated before, immediately after, and 20 min after stretching. Stretching did not affect balance ability in the open-eye condition. After stretching, the sway area was significantly reduced in the closed-eye condition (*p* < 0.05). After stretching, the reach distance of dynamic balance ability increased significantly (*p* < 0.05). The results show that plantar flexor stretching can positively affect balance ability. Therefore, plantar flexor stretching should be considered a rehabilitation method to improve balance.

## 1. Introduction

Balance is defined as the ability to maintain the center of mass within a base of support [1,2], and maintenance of balance is considered an essential factor for people to perform functional movements in daily life and recreational activities [3,4]. Functional movements include actions performed in daily life, such as sitting, standing, and gait, and various work or sports-related movements. These movements are performed and evaluated based on static and dynamic balance [5]. Static balance is the ability of an individual to maintain a certain posture against external and internal stimuli properly, and dynamic balance is the ability to maintain posture when moving weight or performing vigorous activities [5]. People with damage to the musculoskeletal system and nervous system, such as patients with plantar fasciitis or hip fracture, show a decrease in balance ability [6,7] and decreased balance reduces functional movement and increases the rate of falls, a major risk factor for injury and death [8]. Therefore, restoring and enhancing balance ability is essential for patients with musculoskeletal and nervous system injuries, the elderly, and people who require improvement of functional movements due to other problems.

Various forms of rehabilitation, including strengthening and stretching, are used to improve balance ability [9,10,11,12,13,14,15,16]. Multicomponent home-based rehabilitation, including strength, stretching, and balance, taking into account the individual’s home environment, was effective in improving balance and mobility in elderly patients after hip fracture surgery [9]. Similar to the improvement in balance ability through the application of various interventions, resistance strength training has been reported to be effective in improving balance and gait and functional movements, including chair rise and stair climbing [10]. Additionally, stretching is performed to warm-up and improve flexibility in clinic and sports facilities [17,18,19,20,21]. Stretching can cause adaptive changes in the range of motion (ROM), sensory perception, and passive torque by generating biomechanical and physiological changes in the muscles [22,23,24]. These adaptive changes in the lower body, including the calf muscles, can affect postural maintenance strategies and balance ability [25]. However, the effect of stretching on balance ability has yet to be clarified. Therefore, it is important to investigate the effects of postural muscle stretching on balance ability.

Various studies have investigated the relationship between stretching and balance. However, these studies reported inconsistent results on the relationship between the two factors. Martínez-Jiménez et al. [26] reported that static balance ability was improved after static stretching of the lower extremities in healthy adults. On the other hand, Lima et al. [27] reported that static balance ability decreased with an increase in ROM after stretching in young healthy adults. Additionally, Nelson et al. [28] suggested improvement in dynamic balance after stretching exercises, but there was no significant change after nonstretched intervention in nonbalance-trained individuals. Behm et al. [12] reported that muscle activity in healthy male university students after intermittent stretching was not significant, while balance and reaction/movement time was impaired. The difference in these results may be due to differences in the balance ability measurement method used to evaluate the stretching effect between previous studies. However, until now, the effect of different stretching methods on balance ability is unclear.

Therefore, this study aimed to investigate the effect of plantar flexor stretching on balance ability in healthy adults.

## 2. Methods

### 2.1. Participants

Healthy young adults were recruited through bulletin boards, subway advertisements, and social network services. The inclusion criteria for the study were as follows: (1) having not received balance training; and (2) a score of 28 or higher on the Cumberland Ankle Instability Tool. The exclusion criteria were as follows: (1) having not undergone lower extremity or lower back surgery within the past year; (2) pain or disease in the lower limbs or lower back; (3) a difference of more than 1 cm in leg length; (4) limited balance ability or vestibular function; (5) ankle joint being out of normal alignment; and (6) neurological or musculoskeletal disorders. A total of 62 people volunteered for the study; of them, 44 participated in the study. Participants signed the research consent form after being informed about the study procedures, benefits, and risks of side effects. The study was approved by the Institutional Review Board of Gachon University (number: 1044396-202108-HR-177-01).

The sample size was calculated using G-power software (version 3.1.9.4; Heinrich Heine University, Dusseldorf, Germany) [29,30]. The effect size f was set to 0.25, alpha level was set to 0.05, and power was set to 0.8 [31]. Consequently, a sample size of 40 was required. Considering a dropout rate of 10%, 45 participants were required.

### 2.2. Procedure

**Figure 1:**
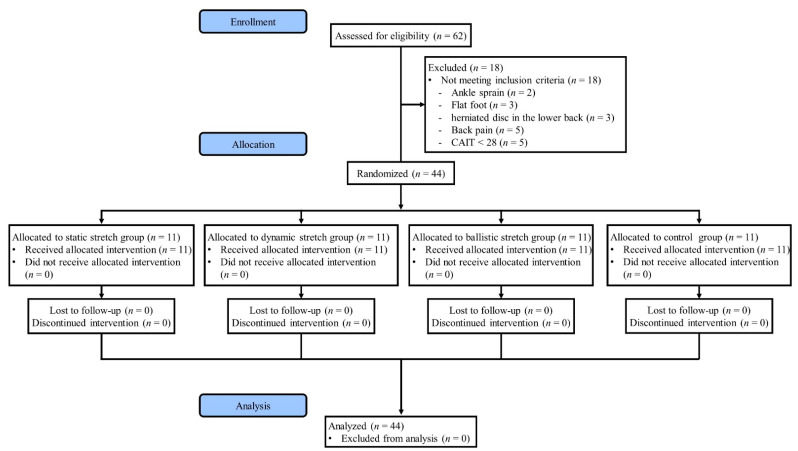
Flow chart in this study.

This study was a single-blind randomized controlled trial. A flow chart in this study is shown in Figure 1. Before the participants were assigned to groups, their general characteristics (gender, age, height, weight, dominant foot, and leg length) were recorded. The dominant foot was defined as that used more than twice during the following actions: (1) kicking a ball; (2) climbing a step box; and (3) stepping on a target. The participants were stratified by gender and dominant foot and divided into four blocks. They were then randomly assigned to one of four groups (static stretch group, dynamic stretch group, ballistic stretch group, and control). Assessments were performed at three time-points (pre-intervention, post-intervention, and 20 min after the intervention). All interventions were performed by physical therapists with more than three years of clinical experience. All assessments were performed by a separate researcher who did not participate in the intervention.

### 2.3. Intervention

All stretching groups repeated four 45 s stretches with a 15 s rest between sets [32,33]. The control group was allowed to sit on a chair and rest for the same duration as the intervention time in the stretching group (4 min).

#### 2.3.1. Static Stretching

**Figure 2:**
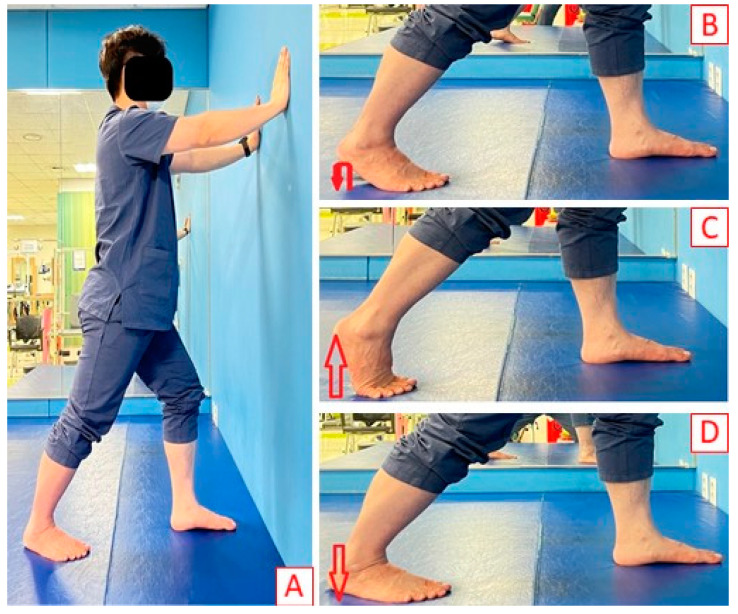
**Stretching position.** (**A**). Static stretching. (**B**). Ballistic stretching. (**C**). Dynamic stretching: raising the heel. (**D**). Dynamic stretching: lowering the heel.

The participants placed both hands on the wall in front of them and their dominant leg one step back (Figure 2A). They were then asked to shift their weight forward while keeping the heel of the back leg on the floor. After shifting their weight until just before uncomfortable sensations occurred, they were asked to maintain their posture.

#### 2.3.2. Ballistic Stretching

After assuming the same posture as for static stretching, the participants were instructed to move up and down using a rebound at the end of the ROM (Figure 2B). They were instructed to repeat the active movement twice per second (2–5°).

#### 2.3.3. Dynamic Stretching

After assuming the same posture as for static stretching, the participants were instructed to repeatedly raise and lower the heel of the dominant leg (Figure 2C,D). They were instructed to repeat the active movement once per second.

### 2.4. Measurements

The evaluation was performed before and after stretching and 20 min after stretching. All evaluation methods were performed after familiarization with ≥10 min of pre-stretching practice.

#### 2.4.1. Ankle Joint ROM

Passive joint ROM of dorsiflexion of the ankle joint was evaluated using a goniometer with high reliability (intra-class correlation coefficient [ICC], 0.68–0.89). The ROM for dorsiflexion was measured as the angle between the line connecting the fibula head and outer malleolus and the longitudinal axis of the 5th metatarsal bone [34].

#### 2.4.2. Balance Ability

Balance ability was evaluated using static and dynamic balance. Static balance was evaluated using an AMTI AccuSway force plate with high reliability (ICC, 0.91–0.99) [35]. During the static balance assessment, the participants were instructed to: (1) turn the dominant foot about 15° outward; (2) look at the sign at eye level located 2 m in front; (3) cross both hands and fix them in front of the chest; and (4) lift the feet of the non-dominant leg more than 10 cm from the floor. Static balance was measured by perturbation of the center of pressure for 20 s. As for the resulting data, the data for the middle 10 s were used in the analysis. Static balance was repeatedly measured three times each with the eyes open and closed.

Dynamic balance was evaluated using the Y-balance test with high reliability (ICC, 0.82–0.87) [36]. During the dynamic balance assessment, participants were instructed to (1) align the tip of the big toe of the dominant leg with the cross shape marked in the center; (2) lift the non-dominant leg and stand on one leg; (3) fix both hands next to the waist; and (4) reach the non-dominant leg to the maximum extent in three directions (frontal, lateral posterior, medial posterior). The measurements were repeated three times for each direction. The resulting data were standardized by dividing the reach by leg length.

### 2.5. Statistics

All statistical analyses were performed using SPSS statistical software (version 25.0; SPSS Inc., Chicago, IL, USA). The Shapiro–Wilk test was used for normality testing. The participants’ general characteristics were compared using the chi-square test and one-way analysis of variance. Balance ability was compared using repeated-measures analysis of variance, and the significance level was corrected using Tukey’s honest significant difference test. Significance was set at α = 0.05.

## 3. Results

### 3.1. Patients’ General Characteristics

Forty-four participants completed the study; there were no dropouts. The participants’ general characteristics are presented in Table 1.

### 3.2. Balance Ability

#### 3.2.1. Static Balance Ability

The static balance ability in the eyes-open condition is shown in Table 2. There was no intergroup difference in static balance ability in the eyes-open condition. The static balance ability in the eyes-closed condition is shown in Table 3. With the eyes closed, the sway area differed significantly before versus after the intervention in the stretching group (*p* < 0.05). The dynamic stretching group showed additional significant changes in sway velocity (*p* < 0.05). However, no significant intergroup differences were noted.

#### 3.2.2. Dynamic Balance Ability

Dynamic balance ability is presented in Table 4. All reach distances in the three directions showed significant improvements in the stretching group (*p* < 0.05). The stretching group showed a significant difference from the control group in the anterior direction (*p* < 0.05). No significant differences were noted between the stretching groups.

### 3.3. Ankle Joint ROM

The ROM of the ankle joints is listed in Table 5. The stretching group showed a significant improvement in ankle joint ROM, and a significant difference from the control group was noted. In contrast, no significant differences were noted between the stretching groups.

## 4. Discussion

This study aimed to investigate the effects of plantar flexor stretching on static and dynamic balance in healthy adults. In all stretching groups, ankle joint ROM, balance ability in the eyes-closed condition, and dynamic balance ability improved after the intervention. Differences among stretching types appeared only in sway velocity in the eyes-closed condition; none were seen in other variables. These results suggest that stretching of the ankle plantar flexors can improve balance ability regardless of type.

Stretching induces biomechanical and physiological changes within the muscles that can alter muscle activation, strength, and kinesthetic awareness [23,24,37]. It can also cause viscoelastic changes in the muscle-tendon unit, resulting in increased muscle length and joint ROM. These factors can affect postural control.

Several studies, including that of Ryan et al. [38], reported improvements in balance after stretching [26,28,39]. In contrast, our results showed no significant change in static balance in the eyes-open condition after stretching. However, our results showed that static balance in the eyes-closed condition and dynamic balance significantly improved. Unlike the Ryan et al. study, which applied stretching to multiple joints and muscles, this study applied stretching to a single muscle. This difference indicates that stretching a single muscle has a weaker effect on balance in a stable situation than stretching multiple muscles. On the other hand, stretching a single muscle can affect balance ability in more challenging situations (increased dependence on somatosensory and dynamic situations).

Depending on the stretching time, this effect can be observed in several ways. Lewis et al. [40] reported that stretching for >2 min did not affect static balance ability. Behm et al. [12] reported a decrease in static balance ability after stretching for more than 2 min in four muscles. Conversely, studies that applied stretching within 1 min reported improvements in static balance [38,39]. This suggests that, due to the viscoelastic characteristics of the muscle, the biomechanical and physiological responses to the muscle appear differently depending on stretching time, which may have different effects on balance ability [41]. Stretching at an appropriate time can prevent unnecessary movement and improve balance by increasing muscle sensory input, sensitivity, and ROM [23,24,42]. Conversely, long-term stretching can lead to a decrease in balance ability by reducing the passive torque of the muscles required to maintain posture [43]. This study applied stretching for 3 min (four 45 s sessions), which is relatively longer than that used in previous studies. However, since the intervention was applied to a single muscle, it did not induce a decline in static balance ability; thus, it is thought to have shown a trend similar to that reported by Lewis [40]. In addition, long-term stretching is thought to increase the ankle joint ROM and improve ankle motion, leading to an improved ability to maintain dynamic equilibrium [42,44].

Previous studies reported differences in balance ability depending on stretching type. However, our study found no difference according to stretching type. As mentioned above, this is considered due to the application of long-term stretching in this study. Since we applied long-term stretching, the muscle elongation and stimulation reached the anatomical limit regardless of stretching type. As a result, no difference was noted between stretching groups in ankle joint ROM or balance ability.

This study confirmed the effect of plantar flexor stretching on balance. However, our study had some limitations. First, because the sample size was small, it is difficult to generalize the study’s results. Second, this study included a limited population. We included only young adults, a high proportion of whom were male. These results may differ for different age groups and gender ratios. Third, the anatomical changes in the muscles and differences in body composition were not evaluated. We indirectly measured muscle changes due to stretching by measuring ankle ROM. As a result, the factors that cause stretching to affect balance other than muscle length and joint angle could not be identified directly. Finally, different stretching times were not applied. The physiological and anatomical effects on the muscles may vary depending on the stretching type and duration. Future studies should analyze changes in balance ability under various intervention conditions.

## 5. Conclusions

The results of this study show that stretching of the plantar flexor muscle improves static balance ability in the eyes-closed condition as well as the dynamic balance ability. Therefore, plantar flexor stretching should be considered a rehabilitation method to improve balance.

## Figures and Tables

**Table 1:** Participants’ general characteristics.

Variable	SG	DG	BG	CG	*p* Value
Gender ^a,^* (M/F)	9/2	9/2	9/2	9/2	1.000
Side ^a,^* (Rt./Lt.)	11/0	10/1	9/2	10/1	0.532
Age ^b,#^ (years)	26.09 ± 1.76	26.27 ± 1.68	26.73 ± 2.15	27.45 ± 3.17	0.507
Height ^b,#^ (cm)	169.82 ± 7.55	174.35 ± 7.98	172.09 ± 7.11	173.64 ± 6.33	0.481
Weight ^b,#^ (kg)	71.64 ± 14.24	77.18 ± 19.76	67.64 ± 11.31	71.34 ± 9.95	0.485
BMI ^b,#^ (kg/m^2^)	24.63 ± 3.27	25.07 ± 4.65	22.69 ± 2.38	23.56 ± 2.13	0.326
CAIT ^b,#^ (score)	29.00 ± 0.89	28.82 ± 0.87	29.45 ± 0.82	29.00 ± 0.89	0.374

BG, ballistic stretch group; BMI, body mass index; CAIT, Cumberland Ankle Instability Tool; CG, control group; DG, dynamic stretch group; F, female; LT., left; M, male; Rt., right; SG, static stretch group. ^a^ Expressed as number of participants; ^b^ Expressed as mean ± standard deviation. * Analyzed using the chi-square test; ^#^ Analyzed using one-way analysis of variance.

**Table 2:** Static balance ability in the eyes-open condition.

Variable		Pre-Intervention	Post-Intervention	Follow-Up	ANOVA *p* Value	Post-Hoc		
						Pre vs. Post	Pre vs. Follow	Post vs. Follow
Sway area (mm^2^)	SG	6.21 ± 2.37	6.91 ± 2.50	7.22 ± 2.60	0.759	0.853	0.759	0.906
	DG	7.40 ± 2.11	7.67 ± 2.61	8.91 ± 3.16		0.977	0.542	0.207
	BG	8.00 ± 3.74	7.01 ± 1.84	6.83 ± 2.32		0.731	0.693	0.969
	CG	8.22 ± 2.56	9.67 ± 7.98	9.42 ± 9.16		0.631	0.774	0.933
Path length (mm)	SG	43.03 ± 9.48	43.24 ± 6.51	42.62 ± 8.20	0.500	0.998	0.991	0.913
	DG	50.66 ± 13.77	50.44 ± 10.01	49.50 ± 9.05		0.998	0.930	0.809
	BG	45.15 ± 7.70	43.38 ± 9.80	42.07 ± 9.48		0.863	0.601	0.667
	CG	52.41 ± 15.19	56.18 ± 15.80	51.93 ± 15.12		0.154	0.815	0.052
Sway velocity (mm/s)	SG	4.30 ± 0.95	4.32 ± 0.65	4.26 ± 0.82	0.648	0.998	0.991	0.906
	DG	5.07 ± 1.38	5.04 ± 1.00	4.95 ± 0.91		0.998	0.929	0.796
	BG	4.51 ± 0.77	4.34 ± 0.98	4.21 ± 0.95		0.857	0.597	0.646
	CG	5.24 ± 1.52	5.62 ± 1.58	5.19 ± 1.51		0.134	0.838	0.093

ANOVA, analysis of variance; BG, ballistic stretch group; CG, control group; DG, dynamic stretch group; SG, static stretch group.

**Table 3:** Static balance ability in the eyes-closed condition.

Variable		Pre-Intervention	Post-Intervention	Follow-Up	ANOVA *p* Value	Post-Hoc		
						Pre vs. Post	Pre vs. Follow	Post vs. Follow
Sway area (mm^2^)	SG	23.02 ± 7.30	18.90 ± 6.04	21.91 ± 6.96	0.257	0.039	0.888	0.294
	DG	25.97 ± 11.03	21.10 ± 7.82	22.19 ± 5.70		0.042	0.263	0.605
	BG	26.05 ± 7.57	22.41 ± 5.48	24.28 ± 6.31		0.046	0.739	0.618
	CG	24.91 ± 7.48	23.28 ± 8.26	24.41 ± 7.93		0.988	0.802	0.837
Path length (mm)	SG	83.54 ± 19.38	82.23 ± 18.60	83.73 ± 21.66	0.826	0.954	0.999	0.888
	DG	97.22 ± 19.91	83.33 ± 14.35	88.17 ± 18.71		0.009	0.120	0.304
	BG	88.27 ± 19.24	83.37 ± 17.77	83.77 ± 16.39		0.523	0.569	0.992
	CG	78.93 ± 29.78	77.14 ± 27.79	75.93 ± 28.11		0.916	0.776	0.925
Sway velocity (mm/s)	SG	8.35 ± 1.94	8.22 ± 1.86	8.37 ± 2.17	0.365	0.954	0.999	0.888
	DG	9.72 ± 1.99	8.33 ± 1.43	8.82 ± 1.87		0.009	0.122	0.305
	BG	8.83 ± 1.92	8.34 ± 1.78	8.38 ± 1.64		0.526	0.574	0.992
	CG	9.14 ± 2.35	8.92 ± 1.99	8.80 ± 2.08		0.879	0.737	0.933

ANOVA, analysis of variance; BG, ballistic stretch group; CG, control group; DG, dynamic stretch group; SG, static stretch group.

**Table 4:** Dynamic balance ability.

Variable		Pre-Intervention	Post-Intervention	Follow-Up	ANOVA *p* Value	Post-Hoc		
						Pre vs. Post	Pre vs. Follow	Post vs. Follow
YANT	SG	69.46 ± 7.07	72.08 ± 7.16 ^a^	72.74 ± 8.17 ^a^	0.016	<0.001	0.002	0.393
	DG	68.01 ± 6.29	70.57 ± 6.52 ^a^	70.21 ± 6.80 ^a^		0.042	0.046	0.412
	BG	68.36 ± 4.45	70.94 ± 3.88 ^a^	70.57 ± 3.72 ^a^		<0.001	<0.001	0.060
	CG	66.58 ± 6.26	67.03 ± 6.30	66.72 ± 6.03		0.744	0.986	0.805
YMED	SG	103.19 ± 11.51	106.88 ± 10.97	106.27 ± 12.12	<0.001	0.001	0.017	0.729
	DG	98.62 ± 7.14	103.80 ± 8.63	104.63 ± 7.70		<0.001	<0.001	0.563
	BG	99.58 ± 8.22	105.25 ± 8.00	107.05 ± 7.03		<0.001	<0.001	0.077
	CG	104.07 ± 11.71	104.14 ± 11.58	104.47 ± 10.54		0.998	0.927	0.910
YLAT	SG	99.27 ± 12.57	103.37 ± 13.15	102.95 ± 13.85	<0.001	<0.001	0.002	0.849
	DG	94.07 ± 10.73	100.06 ± 10.88	100.94 ± 10.73		<0.001	<0.001	0.488
	BG	93.57 ± 9.33	97.84 ± 8.84	98.07 ± 7.97		<0.001	<0.001	0.138
	CG	98.05 ± 15.36	98.57 ± 13.97	99.72 ± 14.14		0.835	0.237	0.297
YSUM	SG	90.64 ± 9.21	94.11 ± 9.08	93.99 ± 10.00	<0.001	<0.001	<0.001	0.969
	DG	86.90 ± 7.36	93.48 ± 8.10	91.93 ± 7.81		<0.001	<0.001	0.294
	BG	87.17 ± 6.69	91.34 ± 6.34	91.90 ± 5.58		<0.001	<0.001	0.342
	CG	89.57 ± 9.57	89.91 ± 9.03	90.30 ± 8.80		0.817	0.523	0.732

ANOVA, analysis of variance; BG, ballistic stretch group; CG, control group; DG, dynamic stretch group; SG, static stretch group; YANT, Y-balance test anterior side; YLAT, Y-balance test lateral-posterior side; YMED, Y-balance test medial-posterior side; YSUM, Y-balance test sum. ^a^ Indicates significant difference from control group (*p* < 0.05).

**Table 5:** Ankle joint ROM.

Variable		Pre-Intervention	Post-Intervention	Follow-Up	ANOVA *p*-Value	Post-Hoc		
						Pre vs. Post	Pre vs. Follow	Post vs. Follow
ROM	SG	18.46 ± 3.77	23.42 ± 3.63 ^a^	22.36 ± 3.50	<0.001	<0.001	<0.001	0.021
	DG	19.70 ± 2.54	24.27 ± 2.31 ^a^	23.33 ± 2.97 ^a^		<0.001	<0.001	0.045
	BG	18.58 ± 3.29	23.21 ± 3.69 ^a^	22.67 ± 3.30 ^a^		<0.001	<0.001	0.332
	CG	18.70 ± 3.96	18.79 ± 3.93	18.67 ± 3.95		0.974	0.997	0.945

ANOVA, analysis of variance; BG, ballistic stretch group; CG, control group; DG, dynamic stretch group; ROM, range of motion; SG, static stretch group. ^a^ Indicates significant difference from control group (*p* < 0.05).

## Data Availability

The data presented in this study are available upon request from the corresponding author.
